# Patient-Reported Control of Asthma, Nasal Polyposis, and Middle-Ear Symptoms in NSAID-Exacerbated Respiratory Disease

**DOI:** 10.3389/falgy.2021.716169

**Published:** 2021-07-15

**Authors:** Anna Suikkila, Lena Hafrén, Annina Lyly, Tuomas Klockars, Riitta Saarinen

**Affiliations:** ^1^Department of Otorhinolaryngology—Head and Neck Surgery, Helsinki University Hospital, University of Helsinki, Helsinki, Finland; ^2^Skin and Allergy Hospital, Helsinki University Hospital, University of Helsinki, Helsinki, Finland

**Keywords:** NERD, AERD, asthma, nasal polyposis, NSAID, aspirin, middle-ear, disease control

## Abstract

Non-steroidal anti-inflammatory drug (NSAID)—exacerbated respiratory disease (NERD) is an adult-onset inflammatory condition of the upper and lower airways. It is characterized by the co-existence of asthma, nasal polyposis, and hypersensitivity to NSAIDs. Over one-fourth of patients also have symptoms of chronic middle-ear infection. The clinical course of NERD is often severe and generally requires multimodal treatment with recurrent surgical measures. Studies presenting the disease burden and subjective symptom control of NERD are limited. In this qualitative questionnaire study, we present the clinical characteristics of asthma, nasal polyposis, NSAID intolerance and possible recurrent or chronic middle-ear infection of 66 confirmed NERD patients treated at our tertiary referral center between January 2016 and May 2017. Additionally, we present the patient-reported disease control of asthma, nasal polyposis, and middle-ear symptoms on a four-category Likert scale. The proportion of NERD patients with recurrent or chronic middle-ear infection was 18%. The proportion of good or very good subjective disease control was 83% for asthma, 58% for nasal polyposis, and 33% for chronic middle-ear infection, if present. Chronic middle-ear infection is common among NERD patients and should more often be recognized as part of the entity. Together with nasal polyposis, chronic middle-ear infection seems to affect patients more than asthma. The patient's perspective of disease control should be considered when planning the interdisciplinary follow-up and treatment of NERD.

## Introduction

Non-steroidal anti-inflammatory drug (NSAID)—exacerbated respiratory disease (NERD) is an adult-onset inflammatory disease of the upper and lower respiratory mucous membranes characterized by the co-existence of asthma, nasal polyposis, and hypersensitivity to NSAIDs. The spectrum was originally described by Samter and Beers (1). It was formerly referred to as aspirin-exacerbated respiratory disease (AERD) or Samter's Triad. As hypersensitivity comprises not only aspirin but also other cyclooxygenase-1 (COX-1) inhibitors, it has been recommended that the term NERD replaces the previously used terms (2).

It has been suggested that the prevalence of NERD in the general population is 0.3–0.9%. The prevalence is higher among patients with asthma (7%), chronic rhinosinusitis with nasal polyps (10%), or severe asthma (15%) (3–5), and is up to 30% among asthmatics with nasal polyposis (6). Most studies have shown that rhinitis is the first symptom. Generally, over about a 5-year time period, a diagnosis of asthma is made, followed by symptoms of NSAID intolerance subsequently leading to a diagnosis of nasal polyposis (7). As many as 26–47% of NERD patients exhibit middle-ear symptoms such as aural fullness, otorrhea, and conductive hearing loss (8, 9). If present, symptoms of otitis media with effusion usually develop following the other manifestations of NERD (10). The clinical course of asthma and nasal polyposis is often severe (2, 3, 11–13). Control of this disease is often challenging, and multimodal treatment with recurrent surgical procedures and systemic corticosteroids are frequently needed (3, 14, 15).

Shared decision-making with patients and adequate symptom-control achievement are listed as key components of precision medicine (16, 17), a concept that aims for individually customized health care and leads to better adherence to treatment, higher patient satisfaction and ultimately, cost savings (17, 18). Studies presenting patients' subjective disease control and disease burden in NERD are limited (19). Furthermore, patients complain about the lack of awareness of NERD among physicians and have even reported conflicts between the health care providers representing different medical specialties who treat them (20). To our knowledge, only one study has assessed the burden and outcome of the otologic symptoms in this patient group. Naples et al. compared otologic-specific SNOT-22 scores before and after endoscopic sinus surgery and aspirin desensitization treatment (21).

In this study, we assessed the patient-reported control of asthma, nasal polyposis, and chronic middle-ear infections in NERD. In addition, we explored the extent of chronic middle-ear infection among NERD patients.

## Method

Helsinki University Hospital (HUH) is a tertiary referral center that provides health care services for 1.67 million citizens. The HUH electronic patient records cover all medical specialties, excluding primary health care.

We conducted a retrospective database search for patients with ICD-10 diagnoses of asthma (J45.0, J45.1, J45.8, J45.9) and nasal polyposis (J33.0, J33.1, J33.8, J33.9) between January 2016 and May 2017. The patient charts were manually reviewed to identify patients with a clinical history and symptoms of NSAID intolerance. Aspirin challenge was not required. A questionnaire, study description, informed consent form and prepaid return envelope were sent to the patients with suspected NERD. The non-respondents were re-approached once. Patients returned the questionnaires between April 2018 and May 2019. We analyzed all the returned questionnaires.

The patients were first asked to confirm that they had diagnoses of asthma, nasal polyposis, and NSAID intolerance. Only patients confirming these conditions were included in the study. The questionnaire was divided into sections covering the medical history of asthma and NSAID intolerance and the medical and surgical history of nasal polyposis. In addition, one section contained questions about a possible adulthood history of chronic or recurrent middle-ear infections. At the end of each section, the respondents were asked to self-assess the current clinical control of asthma, nasal polyposis, and middle-ear symptoms. The disease control was assessed by a four-category qualitative Likert scale. The categories were “very good,” “good,” “poor,” or “very poor.” The middle category of “neither good nor poor” was eliminated to avoid a non-response option (22). The questionnaire is included as Supplementary Material.

The associations between patient characteristics and clinical variables were calculated using Fisher exact tests. A *P*-value of <0.05 was considered statistically significant. Descriptive statistics were used for means, medians, and ranges.

## Results

**Figure 1:**
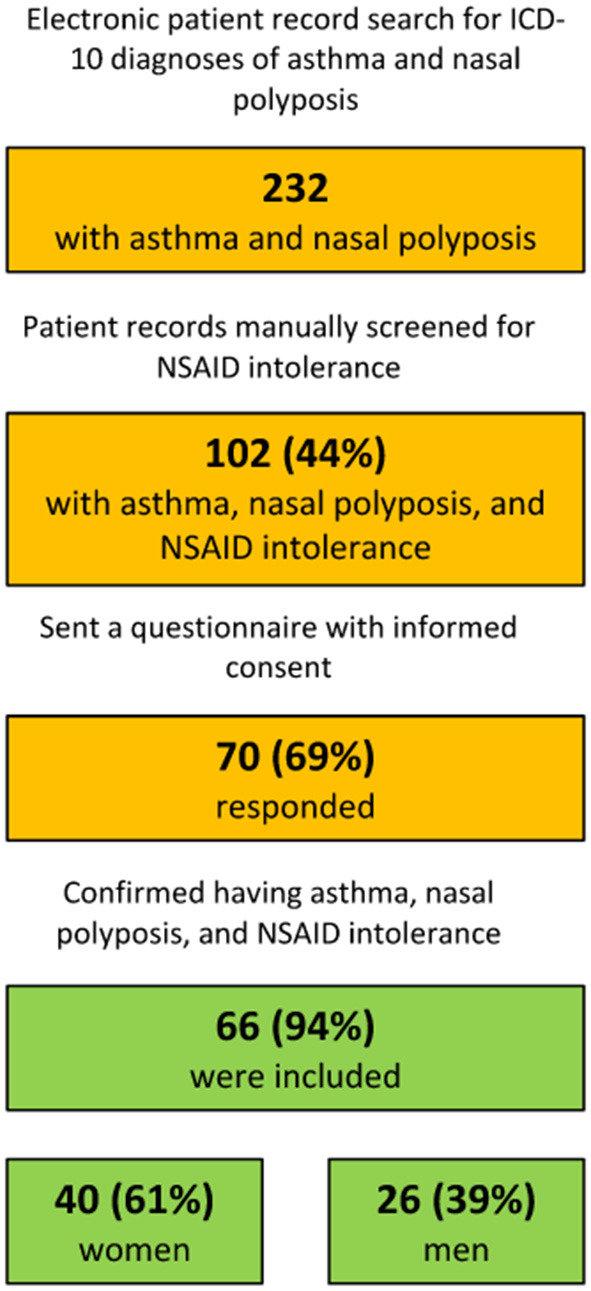
Patient retrieving process.

Figure 1 summarizes the patient retrieval process. A total of 232 patients had ICD-10 diagnoses of both asthma and nasal polyposis. A manual review of the patient charts identified 102 patients (102/232, 44%) with a clinical history of NSAID intolerance. These patients were considered possible NERD patients, and received the questionnaire. Seventy patients (70/102, 69%) responded, and the inclusion criteria (confirmed asthma, nasal polyposis, and NSAID intolerance) were met by 66 respondents (66/70, 94%). Table 1 shows their demographic characteristics in detail. Of the 66 patients, 12 (18%) reported recurrent or chronic middle-ear infection. Nine (9/66, 14%) reported a positive family history of NERD.

**Table 1:** Demographic and clinical characteristics of 66 included patients.

		**Mean**	**Median (range)**	***n* (%)**	**Not answered (%)**
**Demographic data**	Age (years)	52	53 (21-73)	66 (100)	
	Age, men (years)	51	51 (26-71)	26 (39)	
	Age, women (years)	52	54 (21-73)	40 (61)	
**Asthma**	Age at onset (years)	34	33 (3-66)		2 (3)
	Years with phenotype	17	15 (1-48)		
	Set as first diagnosis			38 (58)	
	Inhaled corticosteroids			62 (94)	2 (3)
	Leucotriene modifiers			28 (42)	2 (3)
	Regular peroral corticosteroids			6 (9)	2 (3)
	Peroral corticosteroids as a course in past 5 years	7	5 (1-20)	37 (56)	5 (8)
	Biologic medical therapy			2 (3)	2 (3)
	Positive family history			37 (56)	8 (12)
**Nasal polyps**	Age at onset (years)	36	34 (11-70)		1 (2)
	Years with phenotype	16	12 (1-45)		
	Set as first diagnosis			10 (15)	
	Topical nasal steroids			59 (89)	6 (9)
	Regular peroral corticosteroids			3 (5)	6 (9)
	Peroral corticosteroids as a course in past 5 years	7	13 (1-25)	39 (59)	11 (17)
	Surgical removal of nasal polyps	6	4 (1-70)	63 (95)	2 (3)
	Positive family history			14 (21)	11 (17)
**NSAID intolerance**	Age at onset (years)	35	35 (5-65)		6 (9)
	Years with phenotype	16	15 (0-46)		
	Set as first diagnosis			7 (11)	
	NSAID-related symptoms			64 (97)	2 (3)
	ASA challenge			30 (45)	
	ASA desensitization			23 (35)	
	Successful ASA desensitization			12 (18)	
	Regular peroral ASA 250 mg			5 (8)	
	Positive family history			6 (9)	20 (30)
**Middle-ear symptoms**	Age at onset (years)	35	37 (18-51)	12 (18)	3 (25)
	Women			11 (92)	
	Adulthood myringotomy			9 (75)	
	Adulthood ventilating tubes			7 (58)	
	Adulthood tympanic membrane perforation			6 (50)	
	Current tympanic membrane perforation			5 (42)	
	Bilateral hearing aid			2 (17)	5 (8)

**Figure 2:**
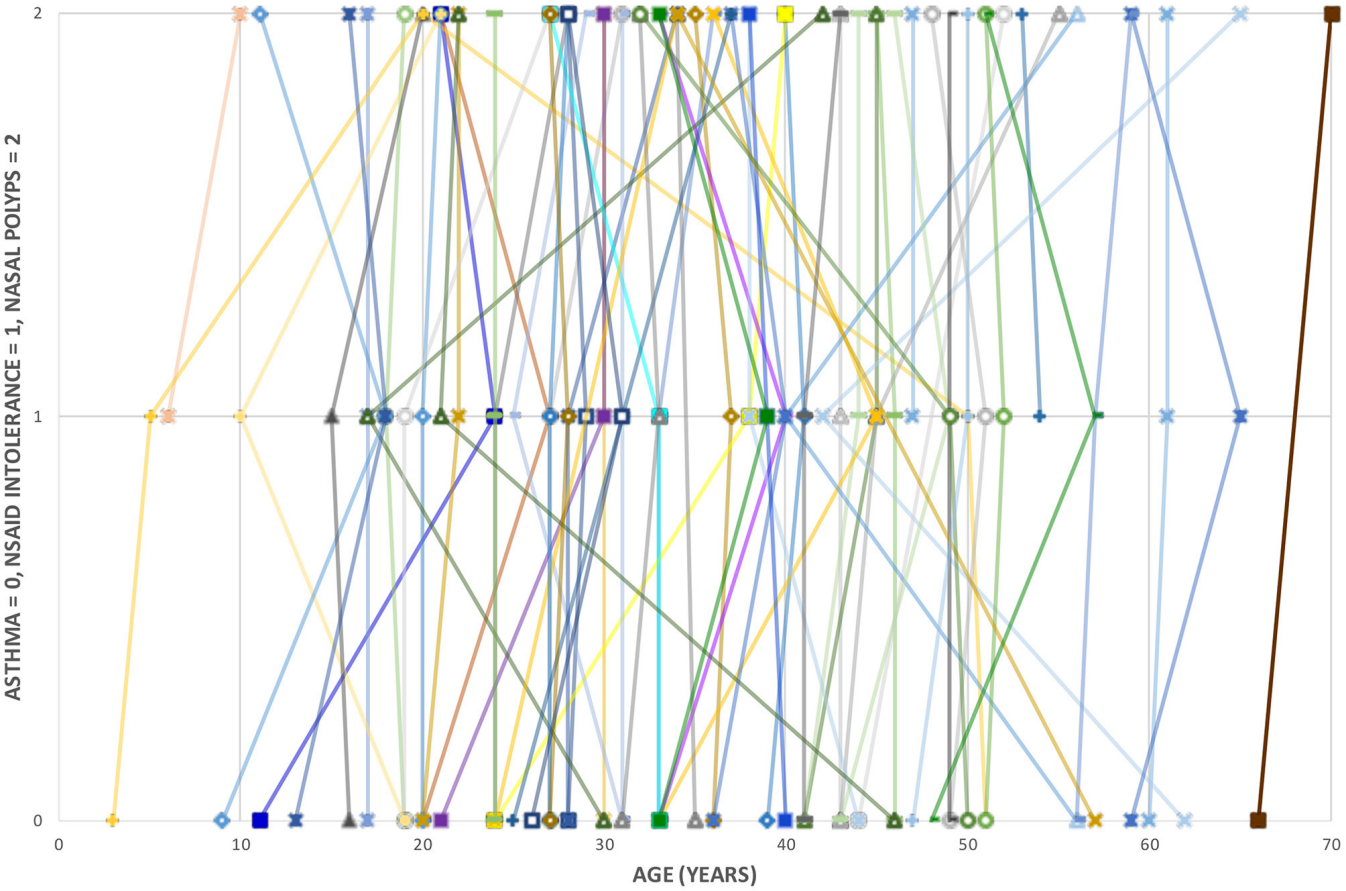
Disease-specific contraction age of 66 included patients.

The diagnoses of asthma, NSAID intolerance, and nasal polyposis were made in a short time span. The median time span was 4 years and the average was 6 years. When taking into account only the mean contraction ages, the first diagnosed condition was asthma, followed by NSAID intolerance and nasal polyposis. However, considerable variation existed in the order and timing of the separate conditions for individual patients. Six patients (9%) received all three diagnoses in the same year. Figure 2 shows the disease-specific contraction time of individual patients.

### Asthma, Nasal Polyposis, and NSAID Intolerance

The median age of onset for asthma was 33. The majority (94%) of the patients used inhaled corticosteroids. Less than half (38%) took leukotriene modifiers regularly, and only two (3%) had biological medical therapy. Two-thirds (64%) had needed peroral corticosteroid treatment either continuously or as a course in the past 5 years. The average number of required corticosteroid courses was seven. More than half of the patients (37/66, 56%) reported a positive family history of asthma.

The median age of onset of nasal polyposis was 34. The majority (88%) used topical nasal steroids regularly. Of these, 23 (35%) used corticosteroid drops and the remainder nasal sprays. Nearly all (95%) had undergone surgical removal of nasal polyps, and the average number of reported operations was six. More than half (59%) had needed a course of oral corticosteroids in the past 5 years, on average seven times. One-fifth (14/66, 21%) reported a positive family history of nasal polyposis.

The median age of onset of NSAID intolerance was 35. The reported aspirin- or NSAID-related symptoms included the worsening of asthma (38%), breathing difficulties (86%), swelling, itching, or irritation of the oral or pharyngeal mucous membranes (42%), skin symptoms (21%), and other symptoms (32%), including nasal congestion and discharge, sneezing, worsening of polyps, eye irritation, nausea, abdominal pain, and swelling of the face. Three patients (5%) reported anaphylaxis. No-one reported skin symptoms exclusively caused by NSAID, which makes NSAID-related urticaria unlikely. Thirty patients (45%) had undergone an aspirin challenge. Twenty-three patients (35%) had undergone aspirin desensitization between 1999 and 2018. Twelve of the desensitized patients (52%) reported relief of symptoms, including the reduced growth of nasal polyps, less nasal congestion and discharge, and less shortness of breath and asthma-related symptoms. Two of the challenged but not desensitized patients reported anaphylaxis on NSAID exposure. Aspirin treatment (250 mg) was used by five patients (8%) at the time of inquiry, which is less than half of those who had undergone successful desensitization with relief of their symptoms. Less than one tenth (6/66, 9%) reported a positive family history of NSAID intolerance.

Table 1 presents detailed clinical characteristics of asthma, nasal polyposis, and NSAID intolerance.

### Middle-Ear Symptoms

About one-fifth (12/66, 18%) of the patients reported recurrent (more than twice a year) or chronic (more than 2 months) adult-onset middle-ear infection. The median age of onset was 37 years. Almost all (11/12, 92%) of the ear-symptomatic patients were women (*p* = 0.0205). The number of infections in the past 5 years varied from two to over ten. The majority (9/12, 75%) had undergone adulthood myringotomy from one to five times. Over half (7/12, 58%) had had tympanostomy tubes inserted from one to eight times in adulthood. Six patients (50%) reported a history of adulthood tympanic membrane perforation and five reported a current tympanic membrane perforation. Two of the middle-ear symptomatic patients (2/12, 17%) had hearing aids, whereas two of the remaining non-ear-symptomatic patients (2/54, 4%) had hearing aids, all bilateral.

### Disease Control

The majority (55/66, 83%) reported the disease control of asthma they had experienced to be good or very good. Only 12% (8/66) reported poor or very poor asthma control. Twelve patients (18%) had had asthma for over 30 years, and four of these reported poor disease control.

In contrast to the reports on asthma, only slightly over half (38/66, 58%) regarded polyp control to be good or very good. The corresponding figure for poor or very poor polyp control was 38% (25/66). Compared to asthma control, the difference is statistically significant (*p* = 0.001).

In the recurrent or chronic middle-ear infection subgroup, over half (7/12, 58%) regarded the control of their middle-ear symptoms as poor or very poor, and only one-third (4/12, 33%) as good or very good.

**Figure 3:**
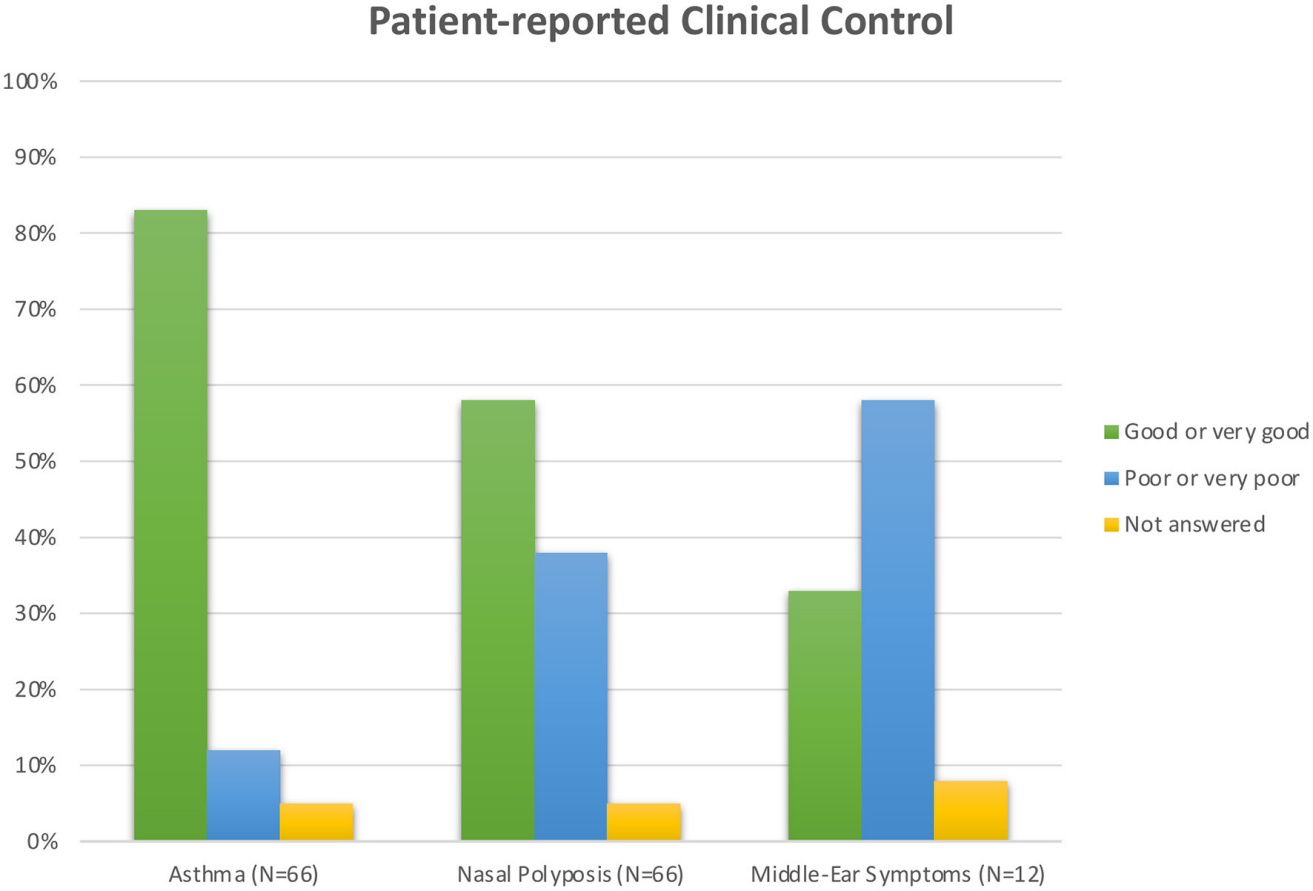
Patient-assessed disease control.

Figure 3 summarizes patient-assessed disease control.

## Discussion

The aim of the treatment of NERD is to achieve adequate control of symptoms and to minimize the effect of the disease on the quality of life (17, 19). This can be achieved by precision medicine: individual care planned together with the patients (16, 17). In this study, we aimed to assess the clinical control of NERD as perceived by the patients.

The females outnumbered the males by 61% in the whole study group and by 92% in the middle-ear infection subgroup. This corresponds to earlier studies (7, 8, 10). The first reported diagnosis was asthma, followed by NSAID intolerance and nasal polyposis. The time span of all three diagnoses was relatively short, the median time span being 4 years. Middle-ear symptoms, if present, developed last. These findings of disease-contraction order, onset age in the fourth decade of life, and diagnosis within a short time span are consistent with earlier data (7, 10). A positive family history of NERD was reported by 14% of our study group, which is significantly higher than the ratio of 1% described earlier (13). This high proportion warrants further study.

The diagnostics and treatment of NERD patients were heterogenous. Less than half were diagnosed with aspirin challenge or treated with aspirin desensitization even though the desensitized patients seemed to also benefit from the treatment. Only a few were on aspirin treatment after desensitization. The use of leukotriene modifiers was reported by less than half of the respondents. Only two were undergoing biological anti-immunoglobulin-E (IgE) treatment for asthma, and none were undergoing biological treatment for nasal polyposis, despite recurrent courses of corticosteroids and numerous surgical procedures for nasal polyp control. Biological monoclonal antibody medication for severe nasal polyposis is a relatively recent therapeutic option (23), and the timing of the data collection is likely to have played a part in the scarce usage of biological medication in our cohort.

Studies presenting the clinical control and disease burden of NERD are limited (19, 20, 24), but have found patients' subjective experience of asthma control and overall quality of life to be better than expected (19, 25). These patients report nasal symptoms as having the greatest impact on their quality of life (24), and this is in line with our findings. Interestingly, the recurrent need for oral corticosteroids in our study cohort indicates that the patients' perceived disease control of asthma might be better than it was objectively assessed to be.

Studies of middle-ear symptoms associated with NERD are scarce and their impact on the patient's quality of life is not well-documented (21). In our findings, 18% of the NERD patients had a chronic middle-ear infection. The proportion in earlier studies has been similar or even higher (8, 9). Of our middle-ear symptomatic NERD patients, the majority had needed a myringotomy or tympanostomy tubes to control their symptoms. Almost half had a current tympanic membrane perforation, indicating chronic inflammation. Furthermore, according to the patients' subjective experiences, the control of middle-ear symptoms was the poorest. In line with Naples et al., we emphasize that chronic middle-ear infection should be considered a part of NERD (21), and that the burden it causes to patients must not be underrated.

This study has some limitations. The data were collected through a database search for ICD-10 diagnosis codes for asthma and nasal polyposis. Thus, we may have missed some patients without a registered diagnosis, and the total number of patients might be higher. Moreover, the data were collected in a university tertiary care center, excluding primary health care, which might have led to the relatively high 44% prevalence of NERD among our asthma and nasal polyposis patients compared to the 30% prevalence described earlier (6).

The study design relied on information collected from the patients, thus posing the risk of reporting bias. Furthermore, we did not require a positive aspirin challenge for the diagnosis of NERD. Instead, intolerance was based on registered symptoms from aspirin intake on patient charts and the patient's subjective confirmation of intolerance. However, previous studies have shown that the clinical history of NSAID intolerance appears to be reliable (2, 5, 26).

Recently, some studies have shown that the type of inflammation varies between NERD patients (27, 28). Due to the study design, we did not register blood eosinophilia, prick test or serum immunoglobulin E (IgE) positivity. Thus, we did not distinguish between the subtypes of asthma or nasal polyposis. In addition, we did not register any other anatomic or functional nasal conditions apart from nasal polyposis. These factors might have affected the patient-perceived disease control.

NERD is a chronic inflammatory systemic condition and the diagnoses of upper and lower respiratory tract disorders are often made close to each other, albeit usually on separate occasions and by different specialists. There is a risk of missing the overall picture of a patient's condition, leading to a delay in the diagnosis of NERD. This is harmful, considering the multimodal therapy and commonness of chronic middle-ear infection and the interventions required to control nasal and middle-ear symptoms. Lack of structured care recently emerged in a comprehensive register study, and patients have also complained about this (20, 29). There is a clear demand for systematic cross-department diagnostics and treatment for this challenging patient group as well as for international guidelines and patient counseling (30). Even more importantly, collaboration between different specialties should be enhanced.

According to our findings, nasal symptoms together with chronic middle-ear infection seem to affect patients more than asthma. Whether this is due to the inadequate use of medication or more severe disease remains to be evaluated. The patient's perspective of disease control should be taken into account when planning the follow-up and treatment of NERD. Middle-ear symptoms, although not an official component of the triad, deserve adequate evaluation and treatment and should be taken into account when considering treatment4 with biologics.

## Data Availability Statement

The datasets presented in this article are not readily available because of patient confidentiality. Requests to access the datasets should be directed to the corresponding author.

## Ethics Statement

The studies involving human participants were reviewed and approved by Helsinki University Hospital Ethics Committee (HUH/3078/2017). The patients/participants provided their written informed consent to participate in this study.

## Author Contributions

LH, AL, TK, and RS contributed to conception and design of the study and revised the manuscript critically for important intellectual content. RS screened manually the electronic patient records. AL drafted the questionnaire. AS and LH organized the database. LH performed the statistical analysis and drafted one picture of the manuscript. AS wrote the first draft of the manuscript. All authors have read and approved the submitted version.

## Conflict of Interest

The authors declare that the research was conducted in the absence of any commercial or financial relationships that could be construed as a potential conflict of interest.
