# Supplementary material for: Patient-Reported Control of Asthma, Nasal Polyposis, and Middle-Ear Symptoms in NSAID-Exacerbated Respiratory Disease
Source: Front Allergy. 2021 Jul 15;2:716169. doi: 10.3389/falgy.2021.716169 (PMC8974741; doi:10.3389/falgy.2021.716169)
Supplement: Supplementary file 1 [file Data_Sheet_1.PDF]

Supplementary material

20.11.2017

Study on clinical characteristics and heredity of Samter's Triad

Questionnaire on Symptoms and Background Data

Name
Date of birth
Date of response

Please answer the following questions as exactly as possible:

1. Have you been diagnosed with any of the following conditions?

Asthma	Yes	No
ASA or aspirin allergy	Yes	No
Nasal polyps	Yes	No

If you answered no to one of the previous conditions, no further responses are needed. Please return the signed questionnaire in the attached envelope.

2. In which year were you diagnosed with asthma?

3. What medication do you currently take for asthma?

4. How many courses of peroral corticosteroids have you taken for asthma in the past five years?

5. How would you rate your current asthma control?

    a. Very good
    b. Good
    c. Poor
    d. Very poor

6. In which year were you diagnosed with nasal polyposis?

7. What medication do you currently take for nasal polyposis?

8. Have you undergone surgery to remove nasal polyps?

Yes No

If you answered yes, how many times?

9. Have your nasal polyps been operated on outside of HUH (Helsinki University Hospital)?

10. How many courses of peroral corticosteroids have you taken for nasal polyposis in the past five years?

11. How would you rate your current nasal polyposis control?

a. Very good
b. Good
c. Poor
d. Very poor

12. In which year were you diagnosed as intolerant of anti-inflammatory painkillers (aspirin, ibuprofen, etc.)?

13. What symptoms do anti-inflammatory painkillers cause you?

a. Worsening of asthma
b. Breathing difficulties
c. Skin symptoms
d. Other, please describe:

14. Have you undergone an aspirin challenge?

Yes No

If you answered yes, where and when?

15. Have you been desensitized to aspirin?

Yes No

If you answered yes, where and when?

Did the desensitization relieve your symptoms?

Yes No

If you answered yes, which symptoms?

16. Have you had adult-onset middle-ear infections (more than once a year or lasting more than two months)?

Yes No

If you answered yes,

a. When was your first adulthood middle-ear infection?
b. How many infections have you had in the past five years?

c. When was your last middle-ear infection?
d. Have you undergone a puncture of the tympanic membrane?

Yes No

If you answered yes, how many times?

e. Have you had tympanostomy tubes inserted?

Yes No

If you answered yes, how many times?

f. Have you had a hole in your tympanic membrane as an adult?

Yes No

g. Do you currently have a hole in your tympanic membrane?

Yes No

17. How would you rate your current ear symptom control?

a. Very good
b. Good
c. Poor
d. Very poor

18. Do you have a hearing aid?

a. No
b. Yes, in one ear
c. Yes, in both ears

19. Do any of your relatives (parents, siblings, children) have Samter's Triad, i.e. asthma and nasal polyposis and intolerance to aspirin?

Yes No

If you answered yes, who (sex and relationship)?

20. Do any of your relatives have an intolerance to aspirin?

Yes No

If you answered yes, who (sex and relationship)?

21. Do any of your relatives have nasal polyposis?

Yes No

If you answered yes, who (sex and relationship)?

22. Do any of your relatives have asthma?

Yes No

If you answered yes, who (sex and relationship)?

23. Do you have siblings?

Yes No

Brother(s) Year(s) of birth
Sister(s) Year(s) of birth

24. Do you have biological children?

Yes No

If you answered yes, please state their sex and year(s) of birth.

25. Do your parents have siblings?

Yes No

If you answered yes to your mother, please state their sex and number.
If you answered yes to your father, please state their sex and number.

26. Was your father born in Finland?

Yes No

If you answered no, which country was he born in?

27. Was your mother born in Finland?

Yes No

If you answered no, which country was she born in?

28. If you know where your grandparents were born, please write it down:

The mother of your father The father of your father

The mother of your mother | The father of your mother

29. May we contact you if further questions arise?

Yes | Telephone number:
No

30. Date | Signature and printed name

Supplementary Material. Questionnaire
